# The NLRP3 inflammasome recognizes alpha-2 and alpha-7.3 giardins and decreases the pathogenicity of *Giardia duodenalis* in mice

**DOI:** 10.1186/s13071-023-05688-2

**Published:** 2023-03-03

**Authors:** Panpan Zhao, Jianhua Li, Xin Li, Jingquan Dong, Xiaocen Wang, Nan Zhang, Shan Li, Min Sun, Xichen Zhang, Zhibang Wang, Min Liang, Ying Li, Lili Cao, Pengtao Gong

**Affiliations:** 1 State Key Laboratory of Zoonosis Research, Ministry of Education, College of Veterinary Medicine, Jilin University, Changchun, 130062 Jilin Province People’s Republic of China; 2 Jilin Academy of Animal Husbandry and Veterinary Medicine, Changchun, 130062 Jilin Province People’s Republic of China; 3 Jiangsu Key Laboratory of Marine Bioresources and Environment, Co-Innovation Center of Jiangsu Marine Bio-Industry Technology, Jiangsu Key Laboratory of Marine Pharmaceutical Compound Screening, Jiangsu Ocean University, Lianyungang, 222005 Jiangsu Province People’s Republic of China; 4 College of Life Science, Jilin University, Changchun, 130062 Jilin Province People’s Republic of China

**Keywords:** *Giardia duodenalis*, Alpha-2 giardin, Alpha-7.3 giardin, NLRP3 inflammasome, Pathogenicity

## Abstract

**Background:**

*Giardia duodenalis* is a parasitic organism that can cause giardiasis, an intestinal infection, particularly prevalent in young children, with clinical symptoms of diarrhea. We previously reported that extracellular *G. duodenalis* triggers intracellular nucleotide-binding oligomerization-like receptor 3 (NLRP3) inflammasome activation and regulates the host inflammatory response by secreting extracellular vesicles (EVs). However, the exact pathogen-associated molecular patterns in *G. duodenalis* EVs (GEVs) involved in this process and the role of the NLRP3 inflammasome in giardiasis remain to be elucidated.

**Methods:**

Recombinant eukaryotic expression plasmids of pcDNA3.1(+)-alpha-2 and alpha-7.3 giardins in GEVs were constructed, transfected into primary mouse peritoneal macrophages and screened by measuring the expression levels of the inflammasome target molecule caspase-1 p20. The preliminary identification of *G. duodenalis* alpha-2 and alpha-7.3 giardins was further verified by measuring the protein expression levels of key molecules of the NLRP3 inflammasome (NLRP3, pro-interleukin-1 beta [IL-1β], pro-caspase-1, and caspase-1 p20), the secretion levels of IL-1β, the level of apoptosis speck-like protein (ASC) oligomerization and the immunofluorescence localization of NLRP3 and ASC. The roles of the NLRP3 inflammasome in *G. duodenalis* pathogenicity were then evaluated using mice in which NLRP3 activation was blocked (NLRP3-blocked mice), and body weight, parasite burden in the duodenum and histopathological changes in the duodenum were monitored. In addition, we explored whether alpha-2 and alpha-7.3 giardins triggered IL-1β secretion in vivo through the NLRP3 inflammasome and determined the roles of these molecules in *G. duodenalis* pathogenicity in mice.

**Results:**

Alpha-2 and alpha-7.3 giardins triggered NLRP3 inflammasome activation in vitro. This led to caspase-1 p20 activation, upregulation of the protein expression levels of NLRP3, pro-IL-1β and pro-caspase-1, significant enhancement of IL-1β secretion, ASC speck formation in the cytoplasm and also induction of ASC oligomerization. Deletion of the NLRP3 inflammasome aggravated *G. duodenalis* pathogenicity in mice. Compared to wild-type mice gavaged with cysts, mice gavaged with cysts in NLRP3-blocked mice displayed increased trophozoite loads and severe duodenal villus damage, characterized by necrotic crypts with atrophy and branching. In vivo assays revealed that alpha-2 and alpha-7.3 giardins could induce IL-1β secretion through the NLRP3 inflammasome and that immunization with alpha-2 and alpha-7.3 giardins decreased *G. duodenalis* pathogenicity in mice.

**Conclusions:**

Overall, the results of the present study revealed that alpha-2 and alpha-7.3 giardins trigger host NLRP3 inflammasome activation and decrease *G. duodenalis* infection ability in mice, which are promising targets for the prevention of giardiasis.

**Graphical Abstract:**

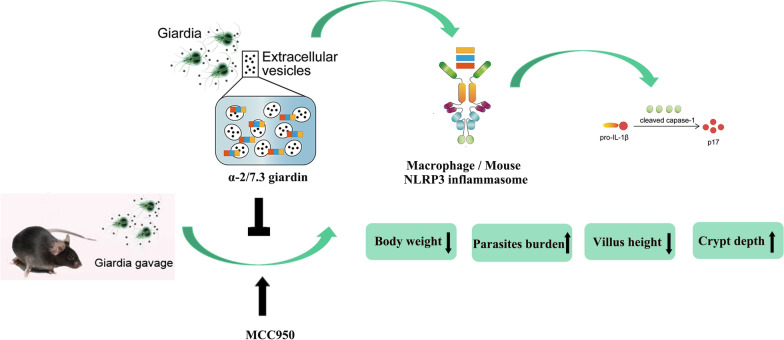

## Background

*Giardia duodenalis*, an extracellular protozoan parasite, colonizes the small intestines and leads to 280 million cases of giardiasis annually, with infection accompanied by diarrhea, especially in young children in developing countries [1]. People are infected through the intake of water or food contaminated with *G. duodenalis* cysts, after which the cysts enter the stomach and are subjected to excystation by gastric acid. *Giardia duodenalis* trophozoites attach to the duodenal epithelial cells and trigger nausea, vomiting, diarrhea, abdominal pain and weight loss. Individuals with immunodeficiency and cystic fibrosis can be easily infected. Infection can also occur through oral and anal sex [2]. Drugs, such as metronidazole, tinidazole and nitazoxanide, are the preferred treatment choices for *G. duodenalis* infections [3]. However, these chemotherapies cause adverse side effects, such as nausea, carcinogenesis and genotoxicity [4]. Consequently, it is necessary to develop more effective preventive strategies against *G. duodenalis* infections.

Inflammasomes, a class of cytosolic complexes of proteins, are part of the innate immune response that aid in the defense against invasion by pathogenic microorganisms and mediate inflammatory responses [5]. Among these inflammasomes, the nucleotide-binding oligomerization (NOD)-like receptor 3 (NLRP3) inflammasome has been widely investigated because it can be recognized by a variety of pathogen/damage-associated molecular patterns (PAMPs/DAMPs), activates the innate immune system and regulates intestinal homeostasis in many inflammatory diseases [6–8]. It is composed of a pattern recognition receptor (PRR) of NLRP3, adaptor apoptosis speck-like protein (ASC) and effector pro-caspase-1 or pro-caspase-11. The NLRP3 inflammasome may function as a host against pathogen invasion, which has been observed in studies on *Neospora caninum* [9], *Paracoccidioides brasiliensis* [10] and *Leishmania spp.* [11], but there are also reports indicating that NLRP3 inflammasome activation restricts the protective immune response and aggravates disease development, such as in helminths [12]. Based on the results of our previous studies, we have reported that extracellular *G. duodenalis* triggers intracellular NLRP3 inflammasome activation and regulates the host inflammatory response by secreting extracellular vesicles (EVs) [13]. However, the role of the NLRP3 inflammasome in *G. duodenalis* infection in vivo remains to be determined.

Giardins were first described as structural cytoskeleton components of *G. duodenalis* and play vital roles in trophozoite movement in the small intestine and in the attachment to epithelial cells. To better adapt to its living environment and enhance its pathogenicity, *G. duodenalis* trophozoites develop unique cytoskeletal structures composed of eight flagella, one median body and one ventral disc [14]. *Giardia duodenalis* trophozoites use their cytoskeleton to move through the upper small intestine, especially the duodenum, and attach to the intestinal epithelium. They continually migrate and re-attach to epithelial cells as well as using the metabolism of cells. Thus, there is a tight connection between their cytoskeletons and virulence. *Giardia duodenalis*-specific giardins are components of cytoskeletal structures [15] and are classified into four classes: alpha-, beta-, gamma- and δ-giardins. There are 21 members of the alpha-giardin family, all of which possess calcium-dependent phospholipid-binding abilities [16]. They can also link the cytoskeleton to the membrane. In individuals with *G. duodenalis-*triggered diarrhea, alpha-giardins are highly expressed and display immunoreactivity during infection [17]. Alpha-1 giardin-based heterologous vaccines play protective roles in fighting against mice giardiasis and are potential candidate antigens for vaccine development [18]. Alpha-8 giardin is located in the plasma membrane and flagella, but not in the ventral disk, and enhances *G. duodenalis* trophozoite motility and growth rate [19]. Alpha-14 giardin adheres to the microtubule structures on the flagella, influencing the vitality of *G. duodenalis* [20]. Alpha-11 giardin is highly abundant throughout its life-cycle, and overexpression of alpha-11 giardin damages *G. duodenalis* itself [21]. However, whether alpha-2 giardin and alpha-7.3 giardin play protective roles against *G. duodenalis* infection, as well as their underlying mechanisms, remains unknown.

In the present study, the recombinant eukaryotic expression plasmids pcDNA3.1(+)-alpha-2 giardin and pcDNA3.1(+)-alpha-7.3 giardin were transfected into primary mouse peritoneal macrophages, and the targets that activated the host NLRP3 inflammasome were subsequently screened. We also evaluated the roles of the NLRP3 inflammasome in *G. duodenalis* pathogenicity, explored whether alpha-2 and alpha-7.3 giardins triggered NLRP3 inflammasome activation in vivo and determined the roles of these two giardins in *G. duodenalis* pathogenicity using mice as a model. Our overall aim was to develop promising targets for the prevention of *G. duodenalis* infection.

## Methods

### Mice handling

Female wild-type (WT) C57BL/6 mice, aged 5-8 weeks, were purchased from LiaoNing Changsheng Experimental Animal Center (Liaoning, China). Mice were given water ad libitum, fed a sterilized diet and housed under a 12/12-h light/dark cycle. Prior to infection, the mice were given antibiotics ad libitum in drinking water supplemented with ampicillin (1 mg/ml), vancomycin (1 mg/ml) and neomycin (1.4 mg/ml) (all purchased from Sangon Biotech, Shanghai, China) [22]. Mice that lost their ability to consume food and water for > 24 h and had lost ≥ 20% of body weight were humanely euthanized by cervical dislocation.

### Preparation of *G. duodenalis *trophozoites, cysts and extracellular vesicles and of primary mouse peritoneal macrophages

The WB *G. duodenalis* trophozoites (American Type Culture Collection, Manassas, USA) were cultured in TYI-S-33 medium supplemented with 12.5% fetal bovine serum (FBS; Every Green, Zhejiang, China) and 0.1% bovine bile (Sigma-Aldrich, St Louis, MO, USA) under microaerophilic conditions. Confluent trophozoites were harvested on ice and passaged at a ratio of 1:4 for further proliferation.

*Giardia duodenalis* cysts were induced as previously described [23], and the trophozoites were collected at the logarithmic growth stage and then diluted to a final concentration of 1 × 10^6^ trophozoites/ml with pH 7.1 induction cyst formation medium (modified TYI-S-33 medium with 0.05% bile concentration). The trophozoites were cultured under anaerobic conditions at 37 °C until the logarithmic stage. The culture medium was replaced with induction cyst formation medium (pH 7.8; modified TYI-S-33 medium with 1% bile concentration), and *G. duodenalis* was cultured at 37 °C for 48–96 h, during which cyst formation was observed under the microscope. After most trophozoites had been induced to form cysts, the culturing mixture was collected and re-suspended in sterile deionized water to lyse the remaining trophozoites. The cysts were counted and preserved at 4 °C for subsequent mouse gavage assays.

*Giardia duodenalis* extracellular vesicles (GEVs) were enriched as previously described [13]. Trophozoites in the logarithmic growth phase were re-suspended in modified TYI-S-33 medium prepared with exosome-depleted FBS (Biological Industries, Beit-Haemek, Israel) at a final concentration of 1 × 10^6^ parasites/ml and cultured for 12 h. GEVs were separated from the culture supernatants by successive centrifugation at 2000 *g* for 10 min, 10,000 *g* for 45 min and 100,000 *g* for 60 min. The pellets were dissolved in phosphate-buffered saline (PBS), quantified using a BCA protein assay kit (Thermo Fisher Scientific, Waltham, MA, USA) and stored at − 80 °C or directly used for further assays.

Primary mouse peritoneal macrophages were prepared as previously described [24]. Briefly, mice (6–8 weeks old) were injected (intraperitoneal [i.p.] route) with 2.5 ml of 2.98% Difco fluid thioglycollate medium (BD, Franklin Lakes, NJ, USA) and fed for 3–4 days. Macrophage suspensions were harvested from mouse peritoneal cavities after euthanasia and centrifuged 3 times at 1000 *g* for 10 min. The harvested cells were detected using the CD11b marker by flow cytometry until the cell purity was > 98%, then added to 6-well cell culture plates (4.5 × 10^6^ cells/well) and cultured in RPMI 1640 medium (Biological Industries) supplemented with 10% FBS (Biological Industries) at 37 °C and 5% CO_2_.

### Construction of recombinant eukaryotic expression plasmids of pcDNA3.1(+)-alpha-2 and alpha-7.3 giardins

RNA was extracted from 1 × 10^7^ trophozoites in 1 ml of TRIzol reagent (Vazyme, Nanjing, China), genomic DNA was extracted from *G. duodenalis* total RNA using MonScript dsDNase (Monad, Wuhan, China) and complementary DNA (cDNA) was synthesized using the MonScript RTIII Super Mix (Monad) according to the manufacturer’s instructions.

The CDS sequence information of the target genes of *G. duodenalis* was obtained from the NCBI GenBank. Specific seamless cloning primers were designed separately for each target gene using Primer 5.0. The forward primers (5ʹ− 3ʹ) were composed of three parts, namely the overlap sequences with *Eco*RV-linearized pcDNA3.1(+) vector (TGGTGGAATTCTGCAGAT) and the initiation codon ATG and GNN (if the first base of the target gene was not G). This was done to enhance the expression efficiency. Additionally, no fewer than 16-bp combinative bases (40–60% GC contents/Tm value around 55 °C) were included. The reverse primers (5ʹ− 3ʹ) were composed of two parts, namely the overlap sequences with *Eco*RV-linearized pcDNA3.1(+) vector (GCCGCCACTGTGCTGGAT) and no fewer than 16-bp combinative bases (excluding the last two bases of the stop codon, such as AA or GA to make the recombinant plasmids express their tag protein). The primer sequences are listed in Table 1 and were synthesized by Comate Bioscience Company Limited (Changchun, China).

**Table 1:** Primer sequences of recombinant pcDNA3.1(+)-alpha-2 and alpha-7.3 giardins

Primer name	Genbank number	Sequence (5ʹ-3ʹ)	Product size (bp)
Alpha-2 giardin	XM_001706906	F:TGGTGGAATTCTGCAGATATGGTTCCGAAGCTATCCCAGA	892
		R:GCCGCCACTGTGCTGGATACTCCCTTAGGCGCCAGA	
Alpha-7.3 giardin	XM_001708403	F:TGGTGGAATTCTGCAGATATGGCTGCAGCGAAGGCTA	886
		R:GCCGCCACTGTGCTGGATACATGACGTGCCAGAGGAC	

The underlined parts were introduced bases to enhance the expression efficiency of recombinant pcDNA3.1(+)-alpha-2 and alpha-7.3 giardins

*F* Forward primer,* R* reverse primer

The targets were amplified using Pfu (Tiangen, Beijing, China) or Ex-taq (Takara Biomedical Technology [Beijing] Co., Ltd., Beijing, China) DNA Polymerase, with the prepared *G. duodenalis* cDNA as the template. The eukaryotic expression vector plasmid pcDNA3.1(+) was linearized with the restriction enzyme *Eco*RV and dephosphorylated using Fast AP (Thermo Fisher Scientific). Both linearized pcDNA3.1(+) fragments and amplified target gene fragments were purified using a DNA Gel Purification Kit (Tiangen) and quantified using a Nanodrop ND-2000 (Thermo Fisher Scientific). The pcDNA3.1(+) fragments and each target gene fragment were recombined using the MonClone Single Assembly Cloning Mix (Monad Biotech Co., Ltd., Suzhou, China) and confirmed using DNA sequencing by Comate Bioscience Company Limited (Changchun, China).

### Transfection of recombinant pcDNA3.1(+)-alpha-2 and pcDNA3.1(+)-alpha-7.3 into primary murine peritoneal macrophages

The endotoxin-free plasmids, pcDNA3.1(+)-alpha-2 and pcDNA3.1(+)-alpha-7.3, were prepared using the SanPrep Endotoxin-Free Plasmid Mini Kit (Sangon Biotech). The concentration was kept above 500 ng/μl to ensure that EDTA in the elution buffer did not interfere with the transfection assays. Primary mouse peritoneal macrophages were cultured in 6-well plates with RPMI 1640 complete medium (Biological Industries) for 12 h, following which the cells were washed in warm PBS 3 times to remove penicillin and streptomycin and then cultured in 2 ml of RPMI supplemented with complete medium. The endotoxin-free plasmids, pcDNA3.1(+)-alpha-2 and pcDNA3.1(+)-alpha-7.3 (2.5 μg), were separately diluted in 125 μl of Opti-MEM reduced serum medium (Gibco, Thermo Fisher Scientific). Next, 5 µl of Lipofectamine 2000 transfection reagent (Invitrogen, Thermo Fisher Scientific) was diluted in 125 μl of Opti-MEM Reduced-Serum Medium. Liposome-DNA complexes were prepared by mixing the diluted endotoxin-free plasmid with Lipofectamine 2000 and allowing the mixture to stand at room temperature for 5 min. The complexes were separately transferred into the cells in each well and mixed slowly. After 4 h, the cell medium was replaced with 2 ml of RPMI 1640 complete medium and the culturing continued for a further 24 h. Fresh cell medium was added to the cells and cultured for different time points as per the assay design.

### Detection of key NLRP3 inflammasome proteins using western blot analysis

Protein samples from supernatants and cell lysates were prepared as previously described [25]. The membrane transfer parameters for pro-IL-1β, pro-caspase-1, caspase-1 p20, NLRP3, β-actin and His-tag were 200 mA/90 min. The primary antibody dilution rates were 1:1000 for targets of interleukin-1β (IL-1β; R&D Systems, Minneapolis, MN, USA), caspase-1 (p20) (Adipogen, Switzerland), and NLRP3 (Adipogen SA, Epalinges, Switzerland) and 1:5000 for targets of His-tag (Amylet Scientific, Wuhan, China) and β-actin (Proteintech, Wuhan, China).

### Determination of ASC oligomerization using disuccinimidyl suberate cross-linking assays

Disuccinimidyl suberate (DSS) cross-linking was performed as previously described [26]. The cells were washed 3 times with cold PBS and fully cracked using a 27-gauge needle in 50 µl of ASC reaction buffer (pH 8.0) containing 25 mM Na_2_PO_4_, 187.5 mM NaCl, 25 mM HEPES and 125 mM NaHCO_3_. The mixture was centrifuged at 5000 *g* for 3 min, and the pellets were cross-linked with 10 µl DSS (25 mM in DMSO) and 40 µl ASC reaction buffer at 37 °C for 30 min. After centrifugation at 5000 *g* for 10 min, the pellets were dissolved in a solution of 40 µl ASC reaction buffer and 10 µl of 6× protein loading buffer (TransGen, Beijing, China), and the solution was then quenched at room temperature for 15 min, followed by boiling for 10 min. The protein samples were then subjected to western blot analysis using primary antibodies against ASC (Wanleibio, Shenyang, China) at a dilution ratio of 1:500.

### Measurement of IL-1β secretion using enzyme-linked immunosorbent assays

The cell culture supernatants were collected and used for the detection of the proinflammatory cytokine IL-1β secretions using the Mouse IL-1 Beta ELISA Kit (Invitrogen, Thermo Fisher Scientific), according to previously described procedures [13]. The OD_450nm_ values were converted to protein concentrations using an IL-1β standard curve.

### Observation of ASC and NLRP3 proteins localization using immunofluorescence assays

Cells coated onto glass coverslips were gently washed 3 times in warm PBS, fixed in tissue cell fixative solution (Biosharp, Beijing, China) for 10 min at room temperature (RT), permeabilized in 0.1% Triton X-100 (diluted in PBS; Biosharp) at RT for 20 min and blocked in 5% bovine serum albumin (in PBS) at RT for 2 h. The cells were then separately incubated with primary antibody against ASC (1:100 dilution) or NLRP3 (1:100 dilution) at 4 °C overnight, and secondary antibody of Cy3-labeled goat anti-rabbit IgG(H+L) (1:400; EarthOx, San Francisco, CA, USA) or FITC-conjugated goat anti-mouse IgG (1:400; Earthox) at 37 °C overnight for 1 h in the dark. Cell nuclei were stained with Hoechst 33258 (10 µg/ml; UE, Suzhou, China) for 5 min and observed under a fluorescence microscope (Olympus Corp., Tokyo, Japan).

### *Giardia duodenalis* cyst infection in WT mice and NLRP3 inflammasome-blocked mice

Mice were divided into four groups (*n* = 7 per group): (i) PBS treatment negative control group (PBS only; 100 µl/mouse PBS by gavage, followed 3 h later by 100 µl/mouse PBS i.p. route daily for 7 days); (ii) MCC950 inhibitor [27] treatment negative control group (100 µl/mouse PBS by gavage, followed 3 h later by 10 mg/kg body weight [BW] MCC950 [in PBS] by i.p. route daily for 7 days); (iii) *G. duodenalis* cyst infection group (1.5 × 10^6^ cysts/mouse by gavage, followed 3 h later by 100 µl/mouse PBS by i.p. route daily for 7 days); and (iv) *G. duodenalis* cyst infection combined with group MCC950 inhibitor treatment group (1.5 × 10^6^ cysts/mouse by gavage, followed 3 h later by 10 mg/kg BW MCC950 i.p. route daily for 7 days). The BW of each mouse was monitored daily, and all mice were euthanized on the 7th day. The harvested duodenum (3 cm long) was cut into small pieces in 1 ml of PBS, the cysts were disrupted in PBS at 4 °C overnight and the number of *G. duodenalis* trophozoites was counted under an optical microscope (Nikon Corp., Japan). Fresh duodenum (1 cm long) was isolated for hematoxylin and eosin (H&E) staining.

### Injection of alpha-2 and alpha-7.3 giardins in WT mice and NLRP3 inflammasome-blocked mice

Mice were classified into two groups: (i) the MOCK control group and (ii) the MCC950 inhibitor group. In each group, there were five treatment methods (*n* = 7/treatment group): (i) the PBS treatment negative control group (PBS only; 100 µl/mouse PBS through intramuscular (i.m.) injection (anterior tibial muscle) [28, 29]; (ii) pcDNA3.1(+) plasmid negative control group (100 µg/mouse DNA through i.m. injection); (iii) *G. duodenalis* cyst infection positive control group (1.5 × 10^6^ cysts/mouse by gavage); (iv) pcDNA3.1(+)-alpha-2 plasmid treatment group (100 µg/mouse DNA through i.m. injection); and (v) pcDNA3.1(+)-alpha-7.3 plasmid treatment group (100 µg/mouse DNA through i.m. injection). After 12 h, the mice in the MCC950 inhibitor group received MCC950 (10 mg/kg BW) i.p. daily for 7 days, whereas the mice in the MOCK group were treated with an equal volume of PBS. Blood samples were collected from the eyeballs of the mice and allowed to stand overnight at 4 °C. Serum samples were separated by enzyme-linked immunosorbent assay (ELISA) to measure the IL-1β levels.

### Immunization of alpha-2 and alpha-7.3 giardins in WT mice infected with * G. duodenalis* cysts by gavage

Thirty-five mice were divided into five groups (*n* = 7/group). Group 1 was the PBS-treated negative control group: mice received 100 µl PBS through i.m. injection, followed by 100 µl PBS by gavage 3 days later. Group 2 was the *G. duodenalis* cyst infection-positive control group: mice were injected with 100 µL PBS and then gavaged with 1.5 × 10^6^ cysts/mouse 3 days later. Group 3 was the pcDNA3.1(+) plasmid immunization combined with the *G. duodenalis* cyst infection control group: mice received 100 µg pcDNA3.1(+) plasmid DNA (i.m.) and were then gavaged with 1.5 × 10^6^ cysts/mouse 3 days later. Groups 4 and 5 were the recombinant pcDNA3.1(+)-alpha-2 giardin plasmid or pcDNA3.1(+)-alpha-7.3 giardin plasmid immunization combined with the *G. duodenalis* cyst infection experiment groups: mice received 100 µg pcDNA3.1( +)-giardin plasmid DNA (i.m.) and were then gavaged with 1.5 × 10^6^ cysts/mouse 3 days later. The BW of each mouse was monitored after *G. duodenalis* cyst gavage. Fresh duodenum was collected for parasite burden measurements and HE staining analysis.

### Histopathological analysis of mice duodenum

Histopathological changes were analyzed according to previously published procedures [30]. Fresh duodenum was fixed in tissue cell fixative solution, embedded in paraffin, cut into 4-μm-thick sections, stained with H&E and analyzed under a light microscope. Representative pathological changes from seven tissue slices from seven independent mice were evaluated by pathologists blinded to treatment and captured at 200× magnification. The villus length and crypt depth were measured according to the method described previously.

### Statistical analysis

The in vitro and in vivo results were obtained from three technical replicates. Graphs were drawn using GraphPad Prism 7.00 (GraphPad Software Inc., La Jolla, CA, USA). Differences between two groups were analyzed by the t-test, whereas differences among ≥ 3 groups were analyzed by one-way analysis of variance (ANOVA) using SPSS software (version 22.0; SPSS IBM Corp., Armonk, NY, USA). The homogeneity of variance of the data was analyzed using the Levene test, followed by a Bonferroni post-hoc test (B). Significance is indicated at *P* < 0.05, *P* < 0.01 and *P* < 0.001 (not significant [n.s.]) (*P* > 0.05).

## Results

### Recombinant eukaryotic expression plasmids of pcDNA3.1(+)-alpha-2 and alpha-7.3 giardins activate the murine macrophage inflammasome

**Fig. 1:**
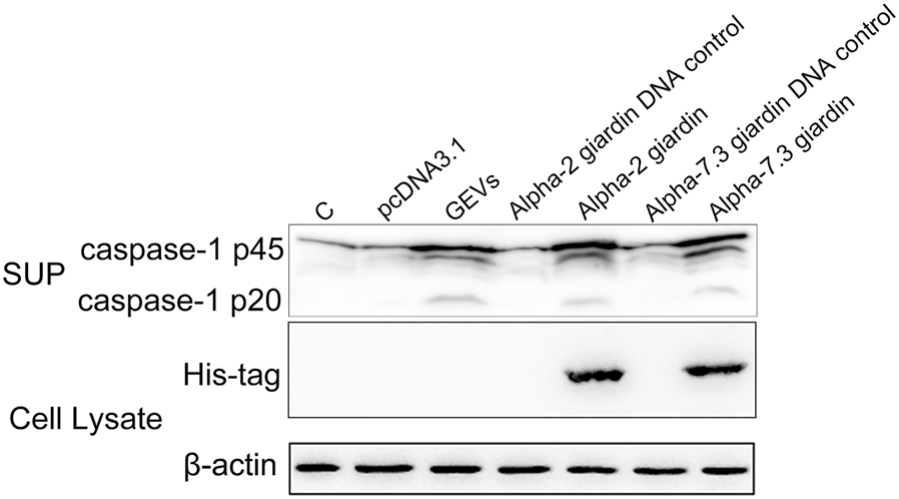
Determination of caspase-1 p20 activated by pcDNA3.1(+)-alpha-2 and alpha-7.3 giardins. The recombinant eukaryotic expression plasmids pcDNA3.1(+)-alpha-2 and alpha-7.3 giardins (above each lane) were transfected into primary mouse peritoneal macrophages, and the medium supernatants were collected at 24 h for measurement of the inflammasome signature protein caspase-1 p20 expression level using western blotting assays. The PBS-only treatment group (lane C) and pcDNA3.1(+)-single treatment group (lane pcDNA3.1) were used as the negative controls, and the GEV treatment group was used as the positive control. The expression of recombinant protein was verified by detecting his-tag in each protein, and the protein bands of the expected size were obtained as alpha-2 giardin (38.2 kDa) and alpha-7.3 giardin (37.2 kDa). GEV, *Giardia duodenalis* extracellular vesicle; pcDNA3.1(+), *Eco*RV-linearized vector; SUP, supernatant

Our previous Kyoto Encyclopedia of Genes and Genomes (KEGG) analysis in GEV proteomics showed that many targets may be involved in activation of the inflammasome signaling pathway [13]. We selected two promising targets, namely the alpha-2 and alpha-7.3 giardins, amplified these molecules and used them to construct the pcDNA3.1(+) eukaryotic expression vector. After sequencing, the recombinant pcDNA3.1(+)-alpha-2 and alpha-7.3 giardin eukaryotic expression plasmids were transfected into primary mouse peritoneal macrophages, and the expression levels of the inflammasome signature protein caspase-1 p20 (activated caspase-1 fragments) were determined to elucidate the key molecules that may trigger inflammasome activation. The results showed that the alpha-2 and alpha-7.3 giardins were able to induce caspase-1 p20 expression, in a manner similar to GEVs. No effect on caspase-1 activation was found in the untreated negative control group (PBS only) and pcDNA3.1(+) plasmid control group (Fig. 1).

### Alpha-2 giardin and alpha-7.3 giardin activate the murine NLRP3 inflammasome in vitro

**Fig. 2:**
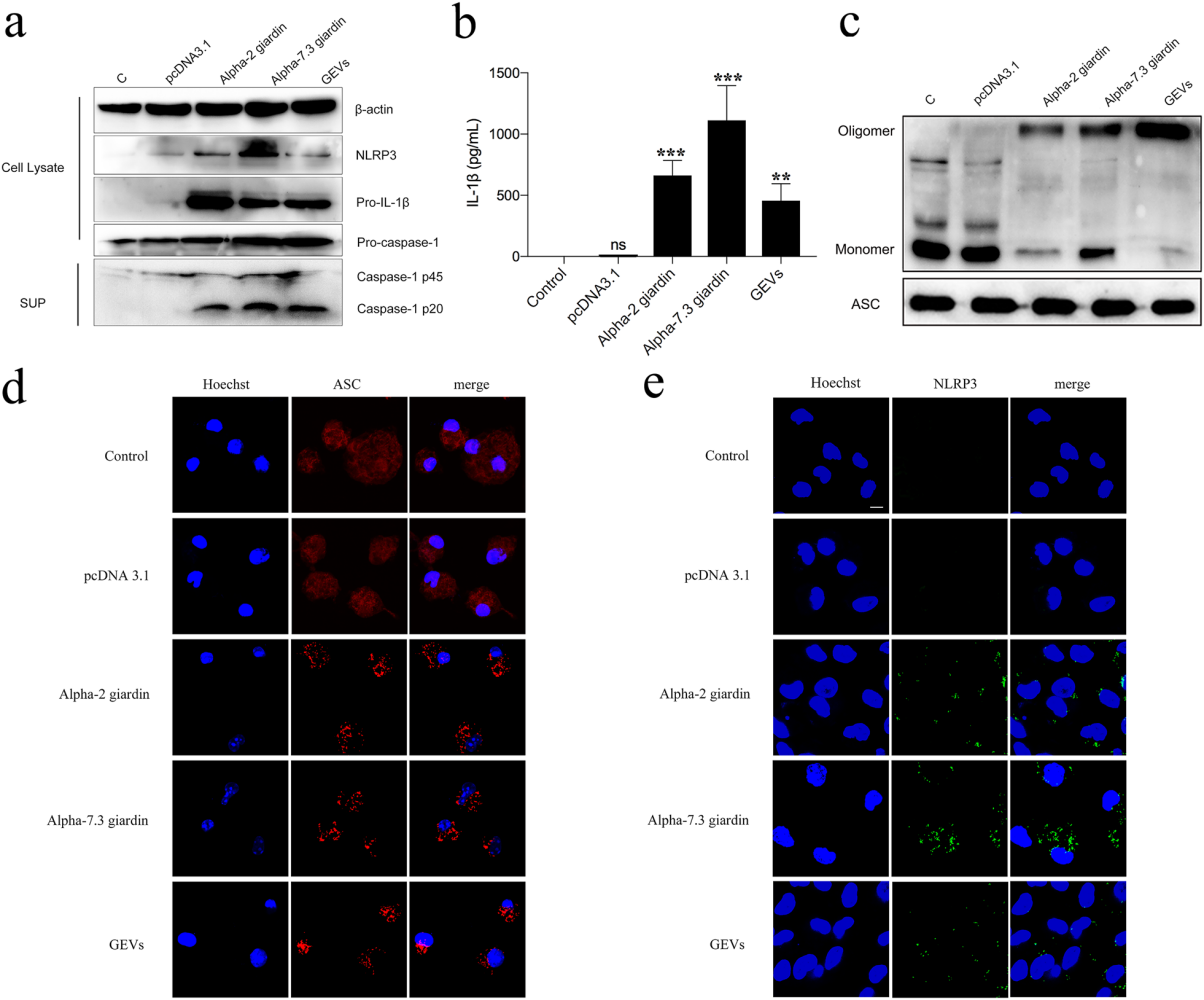
pcDNA3.1(+)-alpha-2 giardin and pcDNA3.1(+)-alpha-7.3 giardin activate mouse peritoneal macrophage NLRP3 inflammasome. The recombinant eukaryotic expression plasmids pcDNA3.1(+)-alpha-2 giardin and pcDNA3.1(+)-alpha-7.3 giardin were transfected into primary mouse peritoneal macrophages and cells or the supernatants were collected at 24 h for analysis of the expression, oligomerization, secretion and location of inflammasome key proteins. The PBS-only group (C) and pcDNA3.1(+) single treatment group were used as the negative controls, and the GEV treatment group was used as the positive group. **a** NLRP3 inflammasome key proteins, including NLRP3, pro-IL-1β, pro-caspase-1 and caspase-1 p20, were detected using western blotting. **b** IL-1β secretion levels in the supernatants were detected using an enzyme-linked immunosorbent assay (ELISA) assay. Differences between the control group and experimental groups were analyzed by one-way analysis of variance (ANOVA) using SPSS version 22.0 software. Asterisks indicate a significant difference between groups at **P < 0.01 and ***P < 0.001. **c** The ASC oligomerization levels were determined in the pellets using the DSS crosslinking assay, whereas the ASC levels in the cell lysates were used as a loading control. **d** ASC localization was observed using immunofluorescence. **e** NLRP3 localization was observed using immunofluorescence. ASC, Apoptosis speck-like protein; IL, interleukin; NLRP3, nucleotide-binding oligomerization-like receptor 3; n.s., not significant (*P* > 0.05)

To determine whether alpha-2 giardin and alpha-7.3 giardin induced caspase-1 p20 expression and played roles in host NLRP3 inflammasome activation, recombinant plasmid DNA of pcDNA3.1( +)-alpha-2 giardin and pcDNA3.1(+)-alpha-7.3 giardin were separately transfected into primary mouse peritoneal macrophages, and the expression, location and oligomerization levels of key proteins of the NLRP3 inflammasome were determined. In this experiment, GEVs were used as the positive control group, and the no treatment group (PBS only) or pcDNA3.1(+) plasmid transfection treatment group were set as the negative groups. The results showed that, similar to the GEV treatment group, the recombinant plasmid DNA of pcDNA3.1(+)-alpha-2 giardin and pcDNA3.1(+)-alpha-7.3 giardin led to the upregulation of NLRP3, pro-IL-1β and pro-caspase-1 and the activation of caspase-1 (Fig. 2a). Moreover, these two giardins induced significant secretion of IL-1β (pcDNA3.1: ANOVA, *F*_(4, 10)_ = 1.625, *P* = 0.1000; alpha-2 giardin: ANOVA, *F*_(4, 10)_ = 1.625, *P* = 0.0007; alpha-7.3 giardin: ANOVA, *F*_(4, 10)_ = 1.625, *P* < 0.0001; GEVs: ANOVA, *F*_(4, 10)_ = 1.625, *P* = 0.0047) (Fig. 2b). Most ASC proteins were in the monomer form in the no treatment group or pcDNA3.1(+) plasmid transfection treatment group; in contrast, the recombinant plasmid DNA of the pcDNA3.1(+)-alpha-2 giardin or pcDNA3.1( +)-alpha-7.3 giardin groups or positive control GEV treatment group underwent ASC oligomerization and behaved as the oligomer form (Fig. 2c). These preliminary data suggest that alpha-2 giardin and alpha-7.3 giardin were able to induce NLRP3 inflammasome activation. Subsequent immunofluorescence studies aimed at localization of ASC and NLRP3 revealed that in the negative control group, ASC proteins were scattered throughout the cytoplasm and appeared as speck-like signals after stimulation of the pcDNA3.1(+)-alpha-2 giardin or pcDNA3.1(+)-alpha-7.3 giardin groups or positive control GEV group (Fig. 2d). In the negative control and pcDNA 3.1 plasmid treatment groups, no NLRP3 protein signals were detected, whereas dots of fluorescent signals were found in the cytoplasm in response to pcDNA3.1(+)-alpha-2 giardin or pcDNA3.1(+)-alpha-7.3 giardin or GEV stimulation (Fig. 2e). These data further illustrate that *G. duodenalis* alpha-2 giardin and alpha-7.3 giardin activate the NLRP3 inflammasome in primary mouse peritoneal macrophages.

### *Giardia duodenalis* attenuates its pathogenicity by activating the NLRP3 inflammasome in mice

**Fig. 3:**
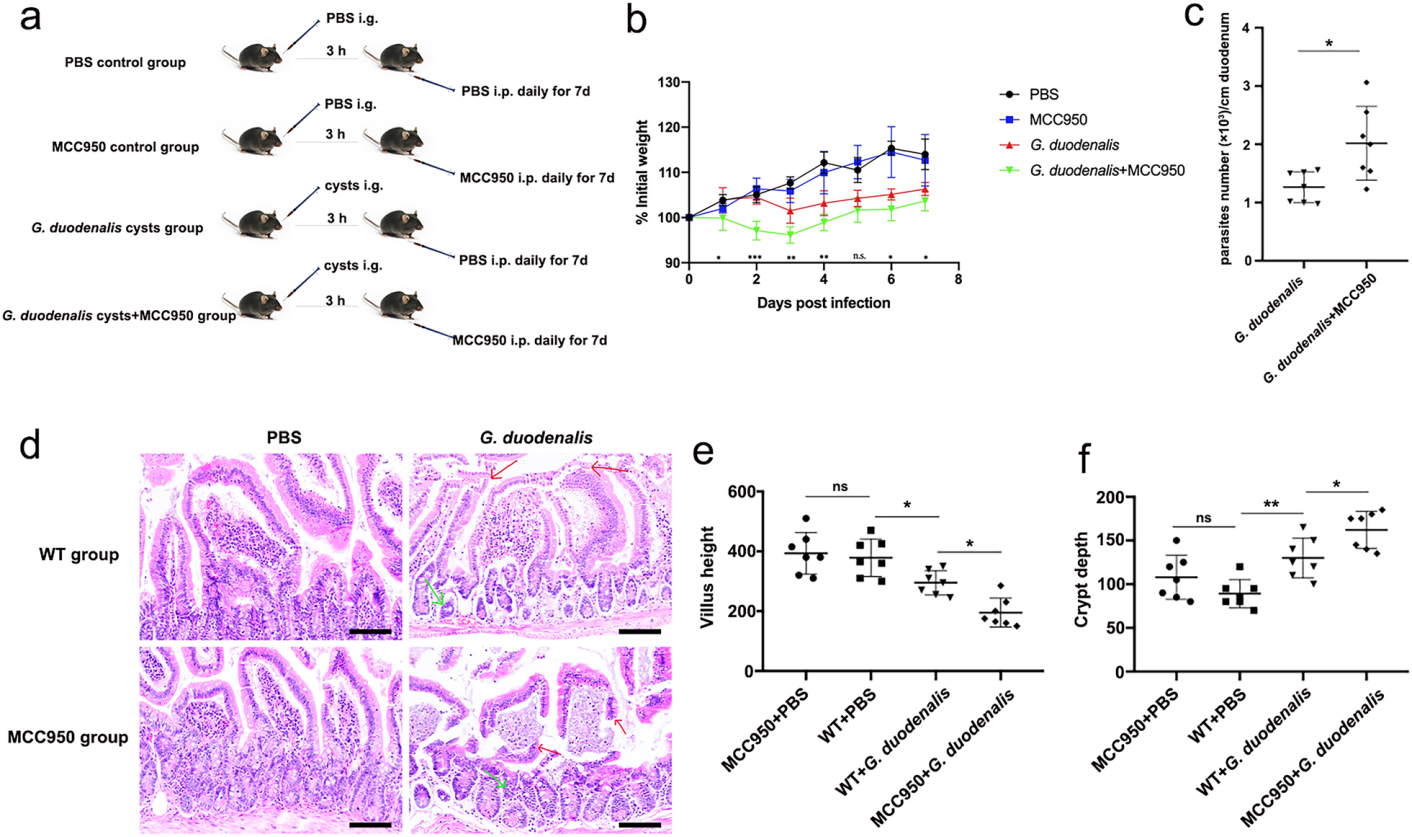
The role of the NLRP3 inflammasome in* Giardia duodenalis *infection. Mice were gavaged (i.g.) with *G. duodenalis* cysts and then received MCC950 treatment (i.p.) or not. The PBS or MCC950 single treatment groups were both used as controls. **a** The experimental groups and treatment scheme. **b** The body weight of mice in each different treatment group was monitored for 7 days. Differences between the *G. duodenalis* infection group and *G. duodenalis* infection + MCC950 treatment group were analyzed by the t-test using SPSS version 22.0 software. Asterisks indicate a significant difference at *P < 0.05, **P < 0.01 or ***P < 0.001. **c** Parasite burden was determined by counting the number of trophozoites in the duodenal lavage fluid. Differences between the *G. duodenalis* infection group and *G. duodenalis* infection + with MCC950 treatment group were analyzed by the t-test using SPSS version 22.0 software. Asterisks indicate a significant difference at *P < 0.05. **d** The duodenum histopathological hematoxylin & eosin (H&E) staining results. The red arrow points to villi lesions; the green arrow points to crypt lesions. Scale bar: 100 μm. **e**, **f** Statistical analysis of villus height and crypt height in mice duodenum. Asterisks indicate a significant difference at *P < 0.05 and **P < 0.01. Results were obtained from 7 independent biological experiments. BW, Body weight; i.g. gavage delivery route; i.p., intraperitoneal delivery route; n.s., not significant (*P* > 0.05); PBS, phosphate-buffered saline; WT, wild type

Both *G. duodenalis* and its secreted GEVs activated the NLRP3 inflammasome and regulated the host inflammatory response in vitro. Thus, the role of the NLRP3 inflammasome in the pathogenicity of *G. duodenalis* is unknown. To explore this problem, we designed experiments with *G. duodenalis* cyst gavage-infected mice and with *G. duodenalis* cyst gavage-infected mice + MCC950 inhibitor treatment, and compared the role of the NLRP3 inflammasome in *G. duodenalis* cyst infection. The detailed experimental scheme is presented in Fig. 3a. Changes in the BW of the mice in the different treatment groups within 7 days after cyst infection were monitored, and the results are shown in Fig. 3b. Compared with the PBS-only treatment group, the results showed that: (i) the BW of mice in the *G. duodenalis* cyst infection group decreased from the third to the seventh day after infection; and (ii) MCC950 inhibitor treatment had no significant effect on the BW of mice. Compared with the single *G. duodenalis* cyst infection group, the BW in the MCC950-treated *G. duodenalis* cyst infection group was decreased at different levels (day 1: ANOVA, *F*_(3, 24)_ = 1.885, *P* = 0.0148; day 2: ANOVA, *F*_(3, 24)_ = 0.4602, *P* < 0.0001; day 3: ANOVA, *F*_(3, 24)_ = 0.8360, *P* = 0.0010; day 4: ANOVA, *F*_(3, 24)_ = 1.683, *P* = 0.0052; day 5: ANOVA, *F*_(3, 24)_ = 0.6497, *P* = 0.0645; day 6: ANOVA, *F*_(3, 24)_ = 5.457, *P* = 0.0175; day 7: ANOVA, *F*_(3, 24)_ = 2.893, *P* = 0.0202). These data suggest that the NLRP3 inflammasome was able to protect mice from significant weight loss in the early stage of *G. duodenalis* infection (2–4 days). Next, we aimed to detect *G. duodenalis* trophozoites in the duodenum lavage fluid, and the results are shown in Fig. 3c. In comparison with the *G. duodenalis* cyst infection group, the number of trophozoites in the duodenum was significantly increased following blocking of the NLRP3 inflammasome (*t*_(12)_ = 2.902, *P* = 0.0133). Staining of the duodenum tissue with HE revealed that compared with the negative control group treated with PBS and MCC950 alone: (i) *G. duodenalis* cyst infection caused duodenal villi damage (ANOVA, *F*_(3, 24)_ = 0.4903, *P* = 0.0488) and crypt atrophy (ANOVA, *F*_(3, 24)_ = 0.4716, *P* = 0.0089); and (ii) the duodenum tissue of mice infected with *G. duodenalis* cysts and treated with the MCC950 inhibitor had no complete duodenum villi structure, and almost all duodenum villi were damaged and necrotic (ANOVA, *F*_(3, 24)_ = 0.4903, *P* = 0.0144) with crypt atrophy and branching (ANOVA, *F*_(3, 24)_ = 0.4716, *P* = 0.0481) (Fig. 3d–f). These results demonstrate that the NLRP3 inflammasome plays a role in reducing *G. duodenalis* pathogenicity.

### Alpha-2 giardin and alpha-7.3 giardin attenuate *G. duodenalis* pathogenicity by activating the NLRP3 inflammasome

**Fig. 4:**
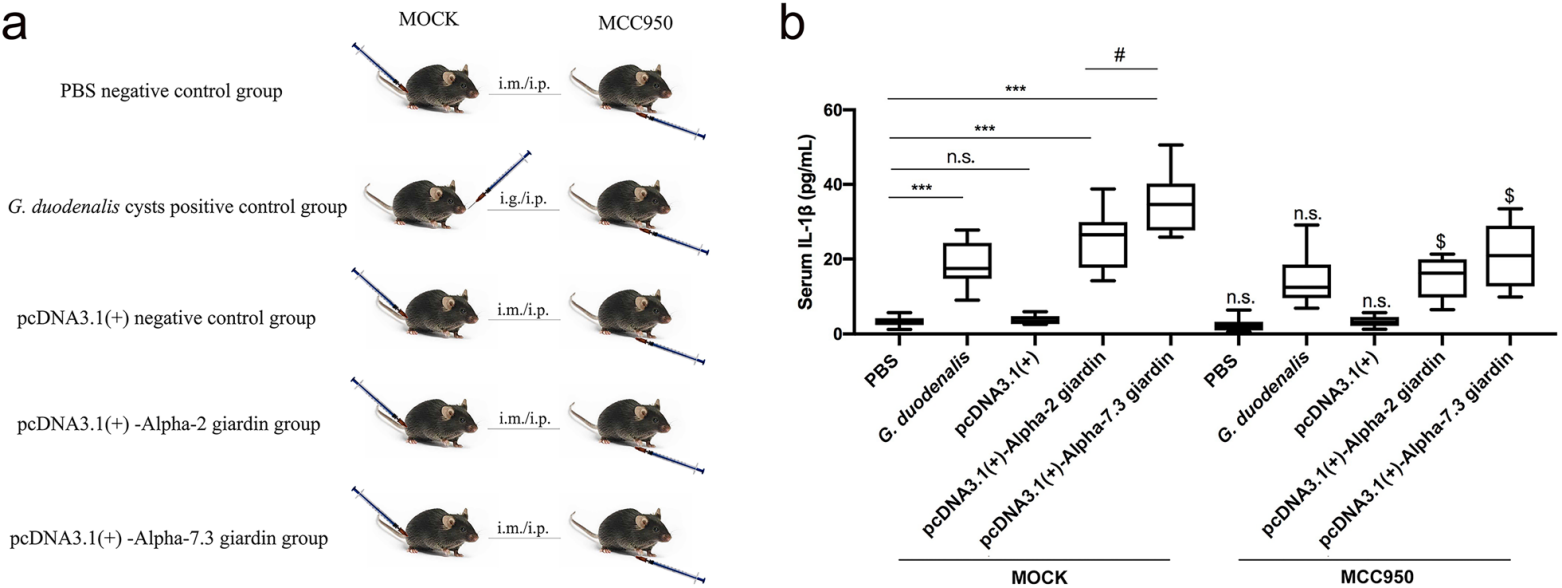
pcDNA3.1(+)-giardins activate host NLRP3 inflammasome in vivo. Mice were immunized (i.m.) with recombinant eukaryotic expression plasmids pcDNA3.1(+)-alpha-2 giardin or pcDNA3.1(+)-alpha-7.3 giardin and then received the MCC950 treatment (i.p.; MCC950 group) or not (MOCK group). The PBS or pcDNA3.1(+) plasmid treatment groups were used as negative controls, and the *G. duodenalis* cyst treatment group was the positive control. **a** The experimental groups and treatment scheme. **b** Mice serum IL-1β levels were detected on the 7th day using ELISA assays. Differences among the groups within the MOCK group were analyzed by one-way ANOVA, while differences between each group in the MOCK group and MCC950 group were analyzed by the t-test using SPSS version 22.0 software. Asterisks indicate a significant difference among treatment groups in the MOCK group at *P < 0.05 and ***P < 0.001; the dollar sign ($) indicates a significant difference at *P* < 0.05 between each group in the MOCK group and the MCC950 group. The results were obtained from seven independent biological experiments. i.m., intramuscular; n.s., not significant (*P* > 0.05)

The secretion of IL-1β is a signature of inflammasome activation. To determine whether *G. duodenalis* alpha-2 giardin and alpha-7.3 giardin activate the host NLRP3 inflammasome in vivo, we used untreated WT mice (MOCK group) and NLRP3 inflammasome-blocked mice (MCC950 inhibitor treatment group), respectively. A detailed experimental scheme is shown in Fig. 4a. Experimental groups consisted of mice receiving the PBS treatment, the *G. duodenalis* cyst gavage treatment, the pcDNA3.1 intramuscular injection and the pcDNA3.1(+)-alpha-2 giardin or pcDNA3.1-alpha-7.3 giardin intramuscular injection treatments. Serum was collected on the seventh day after intramuscular injection of recombinant plasmids, and IL-1β levels in each group were determined. As shown in Fig. 4b, in the MOCK group: (i) in comparison with the PBS group, pcDNA3.1 treatment had no significant effect on IL-1β secretion (ANOVA, *F*_(4, 29)_ = 4.062, *P* = 0.9998), whereas IL-β secretion levels were significantly upregulated in the *G. duodenalis* cyst infection group (ANOVA, *F*_(4, 29)_ = 4.062, *P* = 0.0002); (ii) pcDNA3.1-alpha-2 giardin and pcDNA3.1-alpha-7.3 giardin intramuscular injection significantly upregulated serum IL-1β levels (ANOVA, *F*_(4, 29)_ = 4.062, *P* < 0.0001); (iii) pcDNA3.1-alpha-7.3 giardin induced higher IL-1β secretion levels than those in the pcDNA3.1-alpha-2 giardin intramuscular injection group (ANOVA, *F*_(4, 29)_ = 4.062, *P* = 0.0333). In the MCC950 treatment group, compared with each group in the MOCK group: (i) the secretion levels of IL-1β in the PBS control group and pcDNA3.1 control group were downregulated to some extent after MCC950 inhibitor blocking, but the difference was not significant (PBS: ANOVA, *F*_(9, 58)_ = 3.540, *P* = 0.4912; pcDNA3.1: ANOVA, *F*_(9, 58)_ = 3.540, *P* = 0.5949); (ii) after MCC950 blockade, IL-1β secretion was significantly downregulated in the *G. duodenalis* cyst infection group, pcDNA3.1-alpha-2 giardin group and pcDNA3.1-alpha-7.3 giardin group (*G. duodenalis*: ANOVA, *F*_(9, 58)_ = 3.540, *P* = 0.0120; pcDNA3.1-alpha-2 giardin: ANOVA, *F*_(9, 58)_ = 3.540, *P* = 0.0447; pcDNA3.1-alpha-7.3 giardin: ANOVA, *F*_(9, 58)_ = 3.540, *P* = 0.0164). These results suggest that alpha-2 giardin and alpha-7.3 giardin mediate the activation of the NLRP3 inflammasome in vivo.

**Fig. 5:**
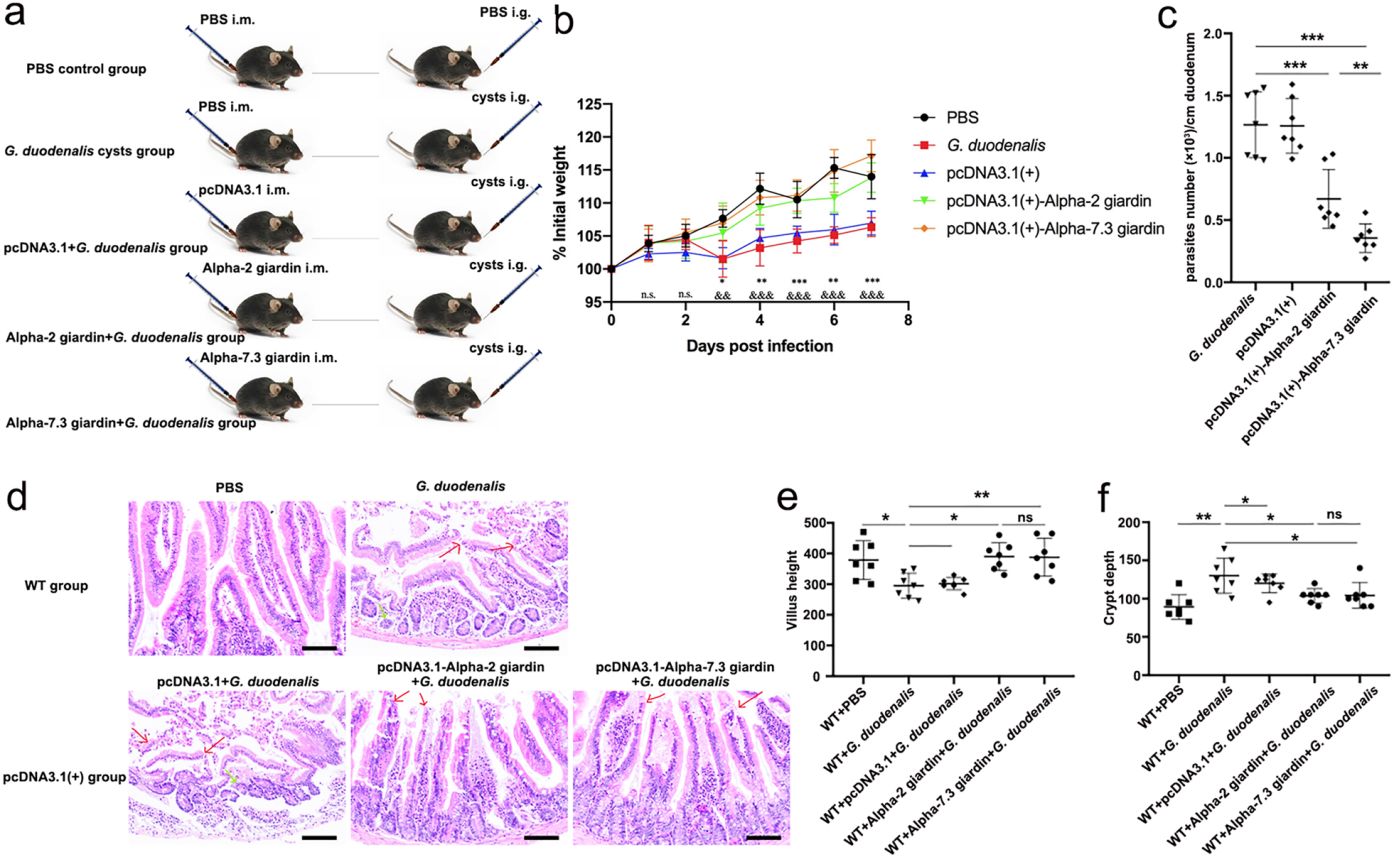
The role of pcDNA3.1(+)-giardins in G. duodenalis infection. Mice were immunized (i.m.) with recombinant eukaryotic expression plasmids pcDNA3.1(+)-alpha-2 giardin or pcDNA3.1(+)-alpha-7.3 giardin and then challenged with *G. duodenalis* cysts (i.g.). The PBS group and pcDNA3.1(+) plasmid + *G. duodenalis* cyst treatment group were used as the negative control groups, whereas the *G. duodenalis* cyst single treatment group was regarded as the positive control group. **a** The experimental groups and treatment scheme. **b** The BW of mice in each different treatment group was monitored for 7 days after challenge. Asterisks indicate a significant difference between each group in the *G. duodenalis* group and pcDNA3.1(+)-alpha-2 giardin group at *P < 0.05, **P < 0.01 and ***P < 0.001; dollar signs ($) indicate a significant difference between each group in the *G. duodenalis* group and pcDNA3.1(+)-alpha-7.3 giardin group at ^$$^*P* < 0.01 and ^$$$^*P* < 0.001. **c** The parasite burden was determined by counting the trophozoites number in 1 ml of the duodenal lavage fluid from harvested duodenum (3 cm long) and presented as parasite number per centimeter duodenum. Differences among the *G. duodenalis* infection group, pcDNA3.1(+)-alpha-2 giardin group and pcDNA3.1(+)-alpha-7.3 giardin group were analyzed by one-way ANOVA using SPSS version 22.0 software. Asterisks indicate significant differences at **P < 0.01 and ***P < 0.001. **d** Histopathological changes in the duodenum. Red arrows point to villi lesions; green arrows point to crypt lesions. Scale bar: 100 μm. **e**, **f** Statistical analysis of villus height (**e**) and crypt height (**f**) in mice duodenum. Differences among each group within Fig. 1d were analyzed by one-way ANOVA using SPSS version 22.0 software. Asterisks indicate significant differences at *P < 0.05 and **P < 0.01. Results were obtained from seven independent biological experiments. n.s., Not significant (*P* > 0.05)

To investigate the effect of alpha-2 giardin- and alpha-7.3 giardin-mediated host NLRP3 inflammasome activation on the infection ability of *G. duodenalis*, we used WT C57BL/6 mice and introduced alpha-2 giardin and alpha-7.3 giardin endotoxin-free plasmids by intramuscular injection, followed 3 days later by gavage with *G. duodenalis* cysts; the mice were then monitored for 7 days. A detailed experimental scheme is shown in Fig. 5a. The BW of each mouse was determined daily, and fresh duodenum tissue samples were collected on the seventh day after gavage to determine the number of trophozoites and to observe histopathological changes. As shown in Fig. 5b, with the prolongation of the feeding stage, the BW of mice in each group increased gradually. The BW of the mice decreased up to the third day after gavage with *G. duodenalis* cysts, then it gradually increased. The activation of the NLRP3 inflammasome induced by intramuscular injection of alpha-2 giardin and alpha7.3 giardin significantly alleviated weight loss in mice (day 1: pcDNA3.1-alpha-2 giardin, ANOVA, *F*_(4, 30)_ = 1.399, *P* = 0.9754; day 1: pcDNA3.1-alpha-7.3 giardin, ANOVA, *F*_(4, 30)_ = 1.399, *P* = 0.9987; day 2: pcDNA3.1-alpha-2 giardin, ANOVA, *F*_(4, 30)_ = 0.3172, *P* = 0.9979; day 2: pcDNA3.1-alpha-7.3 giardin, ANOVA, *F*_(4, 30)_ = 0.3172, *P* = 0.8409; day 3: pcDNA3.1-alpha-2 giardin, ANOVA, *F*_(4, 30)_ = 0.8222, *P* = 0.0262; day 3: pcDNA3.1-alpha-7.3 giardin, ANOVA, *F*_(4, 30)_ = 0.8222, *P* = 0.0083; day 4: pcDNA3.1-alpha-2 giardin, ANOVA, *F*_(4, 30)_ = 0.5620, *P* = 0.0012; day 4: pcDNA3.1-alpha-7.3 giardin, ANOVA, *F*_(4, 30)_ = 0.5620, *P* < 0.0001; day 5: pcDNA3.1-alpha-2 giardin, ANOVA, *F*_(4, 30)_ = 0.9728, *P* < 0.0001; day 5: pcDNA3.1-alpha-7.3 giardin, ANOVA, *F*_(4, 30)_ = 0.9728, *P* < 0.0001; day 6: pcDNA3.1-alpha-2 giardin, ANOVA, *F*_(4, 30)_ = 0.7154, *P* = 0.0012; day 6: pcDNA3.1-alpha-7.3 giardin, ANOVA, *F*_(4, 30)_ = 0.7154, *P* = 0.0006; day 7: pcDNA3.1-alpha-2 giardin, ANOVA, *F*_(4, 30)_ = 0.5369, *P* < 0.0001; day 7: pcDNA3.1-alpha-7.3 giardin, ANOVA, *F*_(4, 30)_ = 0.5369, *P* < 0.0001). Parasite burden was evaluated in the duodenum (Fig. 5c). The number of *G. duodenalis* trophozoites in the alpha-2 giardin and alpha-7.3 giardin injection groups was clearly reduced compared with that in the untreated positive control and pcDNA3.1 empty vector injection groups (pcDNA3.1-alpha-2 giardin: ANOVA, *F*_(3, 24)_ = 1.209, *P* = 0.0002; pcDNA3.1-alpha-7.3 giardin: ANOVA, *F*_(3, 24)_ = 1.209, *P* < 0.0001). Moreover, the protective effect of alpha-7.3 giardin on mice was more significant than that of alpha-2 giardin (ANOVA, *F*_(3, 24)_ = 1.209, *P* = 0.0081). The HE staining results are shown in Fig. 5d–f. Compared with *G. duodenalis-*treated mice and mice injected with *G. duodenalis* combined with pcDNA3.1 empty vector, duodenal tissue lesions in mice injected with alpha-2 giardin and alpha-7.3 giardin were reduced, which was reflected by the increased villi damage (pcDNA3.1-alpha-2 giardin: ANOVA, *F*_(3, 24)_ = 2.466, *P* = 0.0035 or *P* = 0.0068; pcDNA3.1-alpha-7.3 giardin: ANOVA, *F*_(3, 24)_ = 2.466, *P* = 0.0028 or *P* = 0.0055) and reduced crypt atrophy (pcDNA3.1-alpha-2 giardin: ANOVA, *F*_(3, 24)_ = 1.470, *P* = 0.0264 or *P* = 0.0158; pcDNA3.1-alpha-7.3 giardin: ANOVA, *F*_(3, 24)_ = 1.470, *P* = 0.0371 or *P* = 0.0191). These results illustrate that alpha-2 giardin and alpha-7.3 giardin reduce the infection ability of *G. duodenalis* by activating the NLRP3 inflammasome *in vivo*.

## Discussion

*Giardia duodenalis* is a well-known intestinal parasite in humans and other mammals that causes giardiasis. In 2004, it was included in the WHO “Neglected Diseases Initiative” due to its high prevalence rates for 6 years, especially in communities of low socio-economic status [32]. The innate immune system plays a vital role in the immune response to *G. duodenalis* infection. Murine macrophages have been reported to capture and kill *G. duodenalis* by releasing extracellular traps [33]. our previous studies showed that *G. duodenalis*, a noninvasive extracellular parasite, activated the p38 MAPK, ERK, NF-κB p65 and NLRP3 inflammasome signaling pathways of murine macrophages to regulate the host inflammatory response and that this process was enhanced by released GEVs [13, 24]. However, the exact PAMPs in GEVs that are involved in NLRP3 inflammasome-regulated inflammation and the role of the NLRP3 inflammasome in giardiasis remain to be elucidated. To elucidate this two issues, we developed the present study.

The NLRP3 inflammasome is located in the cytoplasm of immune cells and can be activated by various particles, such as uric acid crystals, toxins, bacteria, viruses and parasites. In studies on bacteria, toxins have been identified as key PAMPs that activate inflammasome sensors, leading to inflammation and cell death [34]. Some structurally diverse toxins, such as hemolysins from *Staphylococcus aureus* [35] and *Escherichia coli* [36], hemolysin BL (HBL) from *Bacillus cereus* and non-hemolytic enterotoxin (NHE) from *B. cereus* [37], have been reported to induce NLRP3 inflammasome activation. Studies on viruses have shown that virulence proteins, such as SARS-COV-2 envelope (E) protein [38] and Zika virus NS5 protein [39], are vital PAMPs that are recognized by the NLRP3 receptor. In parasite-related studies, many parasites have been reported to be related to host inflammasome activation, such as *Toxoplasma gondii*, *Trichomonas vaginalis* [40], *Trypanosoma cruzi* [41] and *Leishmania* [42]. *Toxoplasma gondii* virulence-related dense granule proteins GRA35, GRA42 and GRA43 are necessary for the induction of Lewis rat macrophage pyroptosis [43]. In addition, a few studies on *Leishmania* have focused on the single molecules that are involved in NLRP3 inflammasomes, such as parasite membrane lipophosphoglycan [44] or zinc-metalloprotease [45]. Among the annexin-like alpha-giardin gene familly, alpha-1 giardin was shown to be a potential vaccine candidate that provided protection against *G. duodenalis* in a murine model [18]. In our study, we selected the *G. duodenalis* virulence factors alpha-2 and alpha-7.3 giardins, which are unique to* Giardia* but relatively less reported. These two target genes were cloned into the eukaryotic expression system pcDNA3.1(+) vector and used for inflammasome activation determination.

In our mouse model, cleaved caspase fragments acted as the markers of inflammasome activation. Once stimulation occurs, NLRP3 interacts with ASC, recruits pro-caspase and generates active caspase, which cleaves pro-IL-1β and pro-IL-18 into mature IL-1β and IL-18, respectively. Inflammatory caspases (caspase-1, -4, -5, and -11), a family of conserved cysteine proteases, are critical for innate defenses and are involved in inflammation and programmed cell death [46]. Caspase-1 is activated by canonical inflammasomes [47], whereas caspase-4, -5 and -11 are cleaved during the formation of non-canonical inflammasomes [48]. In the present study, we used murine peritoneal macrophages as a model and probed for caspase-1 cleaved capsase-1 p20 as a marker of host NLRP3 inflammasome activation in the *G. duodenalis* infection study. The results showed that many alpha-giardins are responsible for canonical inflammasome activation, which is consistent with the findings of key virulent molecules involved in bacteria and viruses. However, our study was just a preliminary screening, and there are also other molecules that may activate non-canonical inflammasomes, as both canonical and non-canonical inflammasomes have been found to exist in *G. duodenalis* infection, as reported in our previous study [13]. To further determine whether the generated caspase-1 p20 is associated with the NLRP3 inflammasome, we transfected alpha-2 and alpha-7.3 giardins into mouse peritoneal macrophages, detected the protein expression levels of key molecules and the ASC oligomerization level and verified that these two alpha-giardins activated the NLRP3 inflammasome. Our results differ slightly from those of Manko-Prykhoda et al., who reported that stimulation of Caco-2 cells with the *G. muris* or *E. coli* EPEC strain alone could upregulate the fluorescence intensity of NLRP3, ASC and caspase-1, although not significantly, while co-stimulation with *G. muris* and *E. coli* increased the levels of the three proteins [49]. This difference may be due to the different choices of* Giardia* species, cell lines and primary cells. We also carried out in vivo assays using MCC950 in 5-week-old female WT C57BL/6 mice, which are more susceptible to *G. duodenalis*. MCC950 is a potent and selective small-molecule inhibitor of NLRP3 that blocks both classical and nonclassical NLRP3 activation at nanomolar concentrations. MCC950 inhibits NLRP3 activation but does not influence the activation of AIM2, NLRC4 and NLRP1 inflammasomes or the TLR signaling pathways [27]. MCC950 blocks NLRP3 activation but does not inhibit NLRP3 priming, K^+^ outflow, Ca^2+^ influx or the interaction between NLRP3 and ASC; instead, it inhibits NLRP3 inflammasome activation by blocking ASC oligomerization [27]. Therefore, we used MCC950 in an in vivo study to determine the role of the NLRP3 inflammasome following the injection of giardins. Activated caspase-1 p10 cleaves the pro-inflammatory cytokines pro-IL-1β and pro-IL-18 into mature IL-1β and IL-18 [50]. In the present study, serum IL-1β levels from giardin-injected mice with or without MCC950 treatment were used as indicators of whether the NLRP3 inflammasome was activated. As expected, MCC950 treatment significantly downregulated serum IL-1β levels. These data clearly illustrate that *G. duodenalis* alpha-2 giardin and alpha-7.3 giardin were able to activate murine NLRP3 inflammasomes.

Ample evidence has accumulated in the last decade showing that IL-17A is the central regulator of immunity against *G. muris*, inducing IL-17RA signaling, generating antimicrobial peptides and regulating complement activation [51]. However, *Giardia* infection is more likely to occur in young individuals, and it has been reported that *Giardia muris* infection in young mice could not activate the IL-17A response to function in its protective roles [52], which led researchers to look for other immunoregulatory mechanisms in *Giardia* infection. The authors of a recent study reported that *G. muris* could promote the production of antimicrobial peptides and reduce its attachment capacity and the number of trophozoites in the intestinal tract by activating the NLRP3 inflammasome with the aid of *E. coli* EPEC, thus weakening the severity of the *E. coli*-induced disease [49]. The NLRP3 inflammasome is involved in the development of various diseases. Studies have shown that *Pseudomonas aeruginosa* triggers macrophage autophagy to escape cell death and that this process is dependent on the activation of the NLRP3 inflammasome [53]. For* Neospora caninum*, reactive oxygen species-mediated NLRP3 inflammasome activation restricts its replication in hosts, making it a potential therapeutic target [9]. *Paracoccidioides brasiliensis* was found to induce NLRP3 inflammasome activation in mouse bone marrow-derived dendritic cells, leading to the release of the inflammatory cytokine IL-1β, which plays a vital role in host defense [10]. Several *Leishmania* spp., including *L. amazonensis*, *L. major*, *L. braziliensis* and *L. infantum chagasi*, activate NLRP3- and ASC-dependent caspase-1 in macrophage cells, and the infection of *Leishmania* spp. in NLRP3/ASC/caspase-1 gene-deficient mice intensified parasite replication [11]. Zamboni et al. reported that *Leishmania* infection triggered the activation of the NLRP3 inflammasome in macrophages, which limited intracellular parasite replication. Therefore, *Leishmania* may inhibit NLRP3 activation as an avoidance strategy. In in vivo studies, the NLRP3 inflammasome promoted *Leishmania* clearance, but not in tissues [54]. Conversely, in helminth infection studies, activation of the NLRP3 inflammasome suppressed the host's protective immunity against gastrointestinal helminth disease [12]. *Shigella* bacteria is one of the major bacteria responsible for diarrhea worldwide. These bacteria can induce IL-1β production through P2X7 receptor-mediated K^+^ outflow, reactive oxygen species, lysosomal acidification and mitochondrial damage. The NLRP3 inflammasome negatively regulates phagocytosis and the bactericidal activity of macrophages against *Shigella* [55]. Studies on *Plasmodium* demonstrated that AIM2-, NLRP3- or caspase-1-deficient mice infected with *Plasmodium* produced high levels of type-1 interferons and were more resistant to *Plasmodium* infection [56]. However, the roles of alpha-2 giardin and alpha-7.3 giardin in inducing NLRP3 inflammasome activation in mice pathogenicity is unknown.

 In the present study, inhibition of the NLRP3 inflammasome with MCC950 reduced the BW of mice and increased the number of trophozoites in the intestinal lavage fluid, resulting in more severe pathological changes in duodenal tissues. Alpha-2 giardin and alpha-7.3 giardin activated the host murine NLRP3 inflammasome, increased mouse BW, decreased the amount of trophozoites in intestinal lavage fluid and attenuated pathological duodenal lesions. These results suggest that *G. duodenalis* can activate the host NLRP3 inflammasome through alpha-2 giardin and alpha-7.3 giardin and reduce the pathogenicity of *G. duodenalis* in mice. 

In summary, our result show that alpha-2 and alpha-7.3 giardins triggered activation of the host NLRP3 inflammasome and decreased *G. duodenalis* infection ability in mice. As such, these molecules are promising targets for the prevention of giardiasis.

## Data Availability

The data that support the findings of this study are available from the corresponding author at gongpt@jlu.edu.cn

## Abbreviations

ASC: Apoptosis speck-like protein
EVs: Extracellular vesicles
GEVs: *Giardia duodenalis* extracellular vesicles
HBL: Hemolysin BL
IL-1β: Interleukin-1 beta
NLRP3: Nucleotide-binding oligomerization-like receptor 3
NHE: Non-hemolytic enterotoxin
PAMPs/DAMPs: Pathogen/damage-associated molecular patterns
PBS: Phosphate-buffered saline
PRR: Pattern recognition receptor
